# EPR-mediated tumor targeting using ultrasmall-hybrid nanoparticles: From animal to human with theranostic AGuIX nanoparticles

**DOI:** 10.7150/thno.37543

**Published:** 2020-01-01

**Authors:** Guillaume Bort, François Lux, Sandrine Dufort, Yannick Crémillieux, Camille Verry, Olivier Tillement

**Affiliations:** 1 Univ Lyon Université Claude Bernard Lyon 1, CNRS, Institut Lumière Matière, Lyon, France.; 2 NH TherAguix SA, Meylan, France.; 3 Institut Universitaire de France.; 4 Institut des Sciences Moléculaires, CNRS UMR 5255, Université de Bordeaux, Bordeaux, France.; 5 Radiotherapy department, CHU de Grenoble, Grenoble cedex 9, France.; 6 Synchrotron Radiation for Biomedical Research Inserm UA7, University of Grenoble Alps, France.

**Keywords:** theranostic, ultrasmall nanoparticle, EPR effect, clinical translation, AGuIX

## Abstract

Interest of tumor targeting through EPR effect is still controversial due to intrinsic low targeting efficacy and rare translation to human cancers. Moreover, due to different reasons, it has generally been described for relatively large nanoparticles (NPs) (hydrodynamic diameter > 10 nm). In this review EPR effect will be discussed for ultrasmall NPs using the example of the AGuIX® NP (Activation and Guiding of Irradiation by X-ray) recently translated in clinic. AGuIX® NP is a 4 ± 2 nm hydrodynamic diameter polysiloxane based NP. Since AGuIX® NP biodistribution is monitored by magnetic resonance imaging (MRI) and its activation is triggered by irradiation upon X-rays, this NP is well adapted for a theranostic approach of radiotherapy cancer treatment. Here we show that AGuIX® NP is particularly well suited to benefit from EPR-mediated tumor targeting thanks to an ultrasmall size and efficacy under irradiation at small dose. Indeed, intravenously-injected AGuIX® NP into rodent cancer models passively reached the tumor and revealed no toxicity, favoured by renal clearance. Moreover, translation of AGuIX® NP accumulation and retention into humans carrying brain metastases was validated during a first-in-man phase Ib trial taking advantage of easy biodistribution monitoring by MRI.

## Introduction

Cancer treatment remains very challenging to date, mainly due to the high toxicity of the used active drugs. To keep a favourable benefit/risk ratio for the patients, systemic therapies require limiting the amount of administered drugs. Accumulation of active drugs at the tumor site relying on passive targeting is based on the enhanced permeability and retention (EPR) effect, specific for inflammation and tumor microenvironments which display abnormal vasculature. In this case, the intravenously-injected drugs reach and accumulate at the tumor site thanks to the specific biological feature of the tumor environment characterized by wide fenestrations in the blood vessels associated to the absence of lymphatic drainage. EPR-mediated passive targeting has generated a lot of expectation since its initial report in 1986 1, however it is today regarded as controversial because highly dependent on cancer types, cancer development time and even individuals and finally reveals a poor translation from animals to humans 2-4. Due to the uncertainty of EPR effect, the requirement of techniques such as *in vivo* imaging to anticipate accumulation of the drug at the tumor site would be of great interest 5-6. Moreover, EPR effect is expected to induce a drug accumulation of 0.7% of the injected dose (ID) which means that such an amount of targeted drug has to provide a sufficient benefit/risk ratio for the patients 7.

The EPR is mostly described for large size nanoparticles (NPs) with a hydrodynamic diameter (HD) higher than 5 nm exceeding renal clearance threshold. Indeed, large NPs provide the requested properties for drug delivery (high drug loading) and multimodal imaging detection (different types of labels integrated) 8. Moreover, smaller NPs have long been neglected due to some difficulties experienced for their syntheses. The large NPs avoid the extravasation observed for the low-molecular-weight drugs and exhibit an increased plasma half-life, expected to improve accumulation at the tumor site. However, the EPR effect is not restricted to large NPs. Indeed, the pharmacological mechanism for accumulation owing to the EPR effect seems to be a very complex phenomenon based on dynamic feature of blood vessels contributing to modulation of the fenestration size on blood vessel over time 9-10. Contrary to what was expected from theory, ultrasmall NPs (UNPs), with HD lower than 5 nm, are also able to be accumulated and retained at the tumor site by EPR effect. While large NPs are well adapted for carrying a high amount of active ingredients, UNPs provide great advantages when low amount of active ingredients is required or when other therapeutic strategies, such as stimulus-triggered therapy, are planned. Thanks to their ultrasmall size, UNPs could also overcome the difficulty of the large NP to reach the whole tumor environment due to the high interstitial fluid pressure (IFP) induced by the low lymphatic drainage system and the extracellular matrix (ECM) which constitutes a hydrophilic barrier between the blood vessel and the tumor 11. Moreover, UNPs below 5 nm (or below 40 kDa) are eliminated *via* renal excretion and so have much shorter plasma half-life which can be a great advantage to limit systemic toxicity, especially if UNPs are designed to have a higher activation specifically at the tumor site. A recently-described architecture jointly combines some properties of NP and UNP by assembling UNPs into a larger biodegradable NP. This ultrasmall-in-nano approach brings together UNPs within a nanostructure based on matrix (polymer or silica), liposome or layered double hydroxide 12. In this way, the NP allows the transport of therapeutic or imaging agents and exhibits long blood circulation while the UNPs are excreted by the renal pathway after disassembling of the nanostructure. Drug delivery and photothermal therapy with this approach has already been *in vitro* validated. For instance, a silica-based passion fruit-like nanoarchitecture (124.3 ± 23.0 nm HD) with embedded-glutathione-coated gold UNPs (< 6 nm HD) induced hyperthermia cytotoxic effect on a 3D model of pancreatic carcinoma through photothermal therapy upon continuous-wave irradiation at 808 nm 13. This type of nanoarchitecture has also successfully been functionalized with a transferrin-targeting peptide for improving cell internalization 14.

Several types of renal clearable inorganic UNPs have been reported for their efficient tumor targeting owing to the EPR effect 15-17. While silica UNPs 18-19, quantum dots 20 and carbon dots 21 exhibited relatively low accumulation, glutathione 22 or PEG-coated 23 gold UNPs achieved high accumulation and retention at tumor site (2.3 ± 0.9% ID/g and 8.3 ± 0.9% ID/g at 12 h post-injection (p.i.) respectively) similar as nonrenal clearable NPs attributed to their interaction with cancer cells or prolonged plasma half-life due to slow renal elimination. Moreover, the addition of an acidity-targeting function on the glutathione-coated gold UNP using cysteamine-surface modification temporarily increased the accumulation into tumors in a LNCaP acidic prostate cancer model (9.48 ± 2.22% ID/g at 24 h p.i.) 24. Cornell dot (C dot) is one of the few UNPs which have been translated into clinic. This dual-mode UNP is lower than 10 nm HD and is made of silica core with an embedded optical imaging agent (Cy5) and a PEGylated surface modified with ^124^I-chelates for positron emission tomography (PET) imaging and cyclo-RGDY peptides to target the α_n_b_3_ integrin-expressing tumors 25. A pilot clinical trial confirmed the safety and renal elimination (whole-body clearance half-time of 13-21 h) of the C dots in intravenously-injected patients (n=5, 5 mCi) with metastatic melanoma using PET imaging over 72 h p.i. 18. Progressive accumulation and retention was observed in the lesions of several subjects, for instance 0.01% of the injected dose accumulated at 72 h p.i. in a patient with pituitary lesion. This first-in-human study opened the route for a clinical trial to assess real-time image-guided intraoperative mapping of nodal metastases after intravenous (iv) injection of C dot 26, and more recently a clinical study with a new generation of ^89^Zr-labelled C dot (C' dot) for PET detection of malignant brain tumors 27. Recent preclinical investigations revealed efficient surface modification of C' dot with an anti-human epidermal growth factor receptor 2 (HER2) antibody fragment 28. This 7.3 nm (HD) ^89^Zr-labelled UNP exhibited progressive accumulation from 2 h to 24-48 h (8.2 ± 1.1% ID/g and 13.2 ± 2.9% ID/g at 2 h and 24-48 h p.i. respectively) and retention up to 72 h (12.0 ± 1.5% ID/g) at the tumor site after iv injection (200-300 µCi) in a BT-474 xenografted HER2+ breast cancer mouse model. Lower accumulation (~5% ID/g) was observed in the control groups (HER2- cancer model and non-targeted C' dots). Surface functionalisation of C' dot with alpha melanocyte stimulating hormone (αMSH) peptide ligands led to *in vivo* specific targeting of melanocortin-1-receptor-expressing tumors (B16F10 tumor-bearing mouse) even if the accumulation efficacy at the tumor site was lower compared to integrin-targeting strategy (~5.0% ID/g at 24 h p.i.) 29.

**Figure 1:**
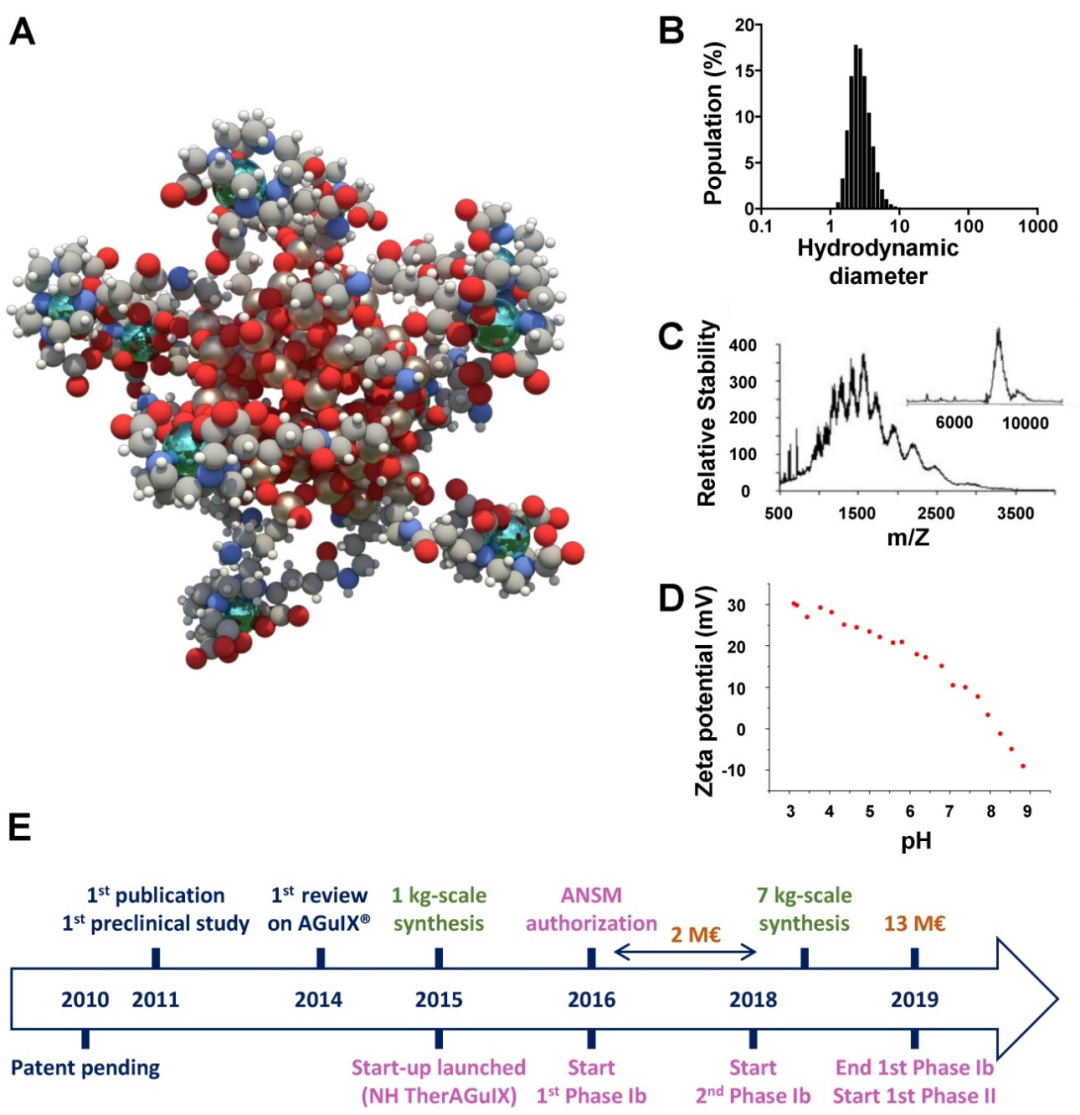
**Description of the AGuIX® NP. A.** Schematic representation of AGuIX® NPs. Gd atoms in green are chelated by DOTAGA (1,4,7,10-tetraazacyclododecane,1-glutaric acid-4,7,10-triacetic acid) ligands grafted to polysiloxane matrix. **B.** HD (4 ± 2 nm) distribution of AGuIX® NPs obtained by dynamic light scattering. **C.** Electrospray ionisation-mass spectrometry measurements on AGuIX® NPs. A mass around 10 kDa was obtained. Inset was obtained by deconvolution with a multiplicative correlation algorithm. **D.** Zeta potential *vs* pH for AGuIX® NPs (isoelectric point around 8.2). **E.** Timescale of the development of AGuIX® NP from bench to bed-side. ANSM: *Agence nationale de sécurité du médicament et des produits de santé*. Adapted with permission from ref 30, copyright 2019 BIR Publications.

Our group has developed a theranostic UNP named AGuIX® NP which is a Gd chelated-polysiloxane matrix based-NP of 4 ± 2 nm HD with a mass around 10 kDa (Figure 1). Thanks to the presence of Gd atoms (~15 wt%), this UNP is detectable by magnetic resonance imaging (MRI) and induces radiosentization during radiotherapy (RT) by increasing the dose effect of about 20% 30. The mechanism of radiosensitization is still not fully understood and several hypotheses are discussed in the literature taking into account physical, chemical and biological approaches 30-31. The currently most accepted hypothesis is a direct or indirect interaction of the high-energy photons with elements of high atomic number (such as the Gd atoms contained in the AGuIX® NPs) through photoelectric effect which induces a local increase of the dose effect by generating a large amount of radicals leading to cell death. The synthesis procedure and characterization of the AGuIX® NP 32, as well as the required key parameters for translation into clinic 30, 33, will not been discussed herein. This review highlights some preclinical studies of AGuIX® NP focusing on its accumulation and retention at the tumor site and reports some preliminary data obtained from NANORAD clinical trial of phase Ib for radiosensitization in patients affected by multiple brain metastases 34-35. We emphasize some important feature to be considered for drug development aiming to reach the tumor site by EPR effect. After iv injection of AGuIX® NPs, MRI allows to monitor real-time accumulation at the tumor site and validates EPR effect efficiency. The AGuIX® NPs which do not reach the tumor site are rapidly washout from the blood thanks to renal filtration due to their ultrasmall size, which prevent any toxicity. MRI monitoring allows to accurately determine the time corresponding to the highest drug concentration at the tumor site, which is also the optimal time for the RT treatment. Here, the low percentage of drug in the tumor compared to the ID is not a problem since the drug is not active except at the irradiated tumor area as demonstrated in healthy rodents 33, 36 and non-human primate models 37-38. As long as the AGuIX® NP concentration at the tumor site is high enough to have a benefit for the patients upon RT, even low accumulation can suit for therapeutic applications.

## Preclinical studies on brain tumors

Three types of central nervous system cancer models have been studied: (i) 9L gliosarcoma (rat) 36, 39-40, (ii) B16F10 brain melanoma metastases (mouse) 41 and (iii) U87MG glioblastoma (mouse) 42-44.

**Figure 2:**
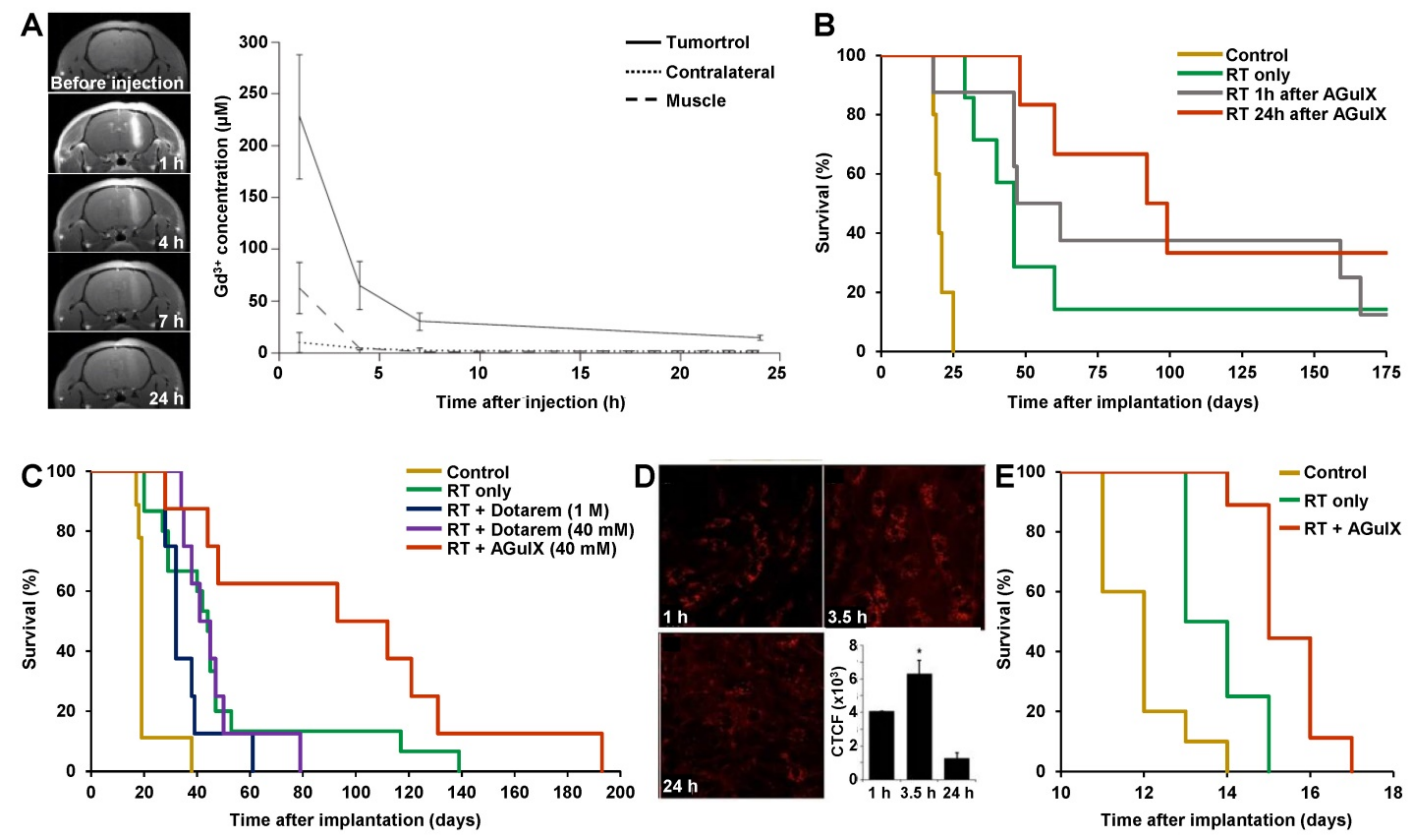
**Studies of AGuIX® NPs on gliosarcoma and brain melanoma metastases rodent cancer models. A.** MRI and Gd quantification in 9L-ESRF-bearing rats. Left panel: the T_1_-weighted MR images were acquired before and 1, 4, 7 and 24 h after iv injection of 1 ml of AGuIX® NPs ([Gd^3+^] = 100 mM) over three rats 14 days after tumor implantation. The pictures show an enhancement of tumor-T_1_ contrast due to the AGuIX® NPs, until 24 h p.i.. Right panel: Gd concentrations issued from T_1_ maps as a function of time elapsed after iv injection (n = 3) in three regions of interest. The results were expressed as the means ± standard deviation. **B.** Survival curve comparison obtained for 9LGS-bearing rats without treatment (yellow curve, n = 5), only treated by microbeam RT (MRT) (green curve, n = 7), and treated by MRT 1 h (grey curve, n = 8) and 24 h (red curve, n = 6) after AGuIX® NP iv injection during 170 days after tumor implantation. **C.** Survival curves of 9LGS-bearing rats without treatment (yellow curve, n = 9), only treated by MRT (green curve, n = 15), and treated by MRT 20 min after an injection of 1 M (56 μL) of Dotarem® (blue curve, n = 8), a unique injection of 40 mM (1.4 mL) of Dotarem® (purple curve, n = 8) or AGuIX® NP (red curve, n = 8), 10 days after tumor implantation. **D.** Intravital two-photon microscopy of labelled AGuIX® NP in subcutaneous B16F10 tumors at 1, 3.5 and 24 h after iv injection, and at the bottom right the corresponding normalized cell fluorescence (CFCT). CFCT was calculated as CFCT = Integrated Density - (Area of selected cell × Mean fluorescence of background reading). **E.** Kaplan-Meier survival curve comparison obtained for brain B16F10 metastases-bearing mice without treatment (yellow curve, n = 10, including 5 mice injected with AGuIX® NPs without radiation exposure), only treated with 7 Gy radiation exposure (green curve, n = 8), and treated with a combination of AGuIX® NPs (10 mg, 3.5 h after iv injection) and 7 Gy radiation exposure (red curve, n = 9) (*p* < 0.001). Adapted with permission from ref 36, 39-41, copyrights 2019 Future Medicine Ltd, Nature Publishing Group, Springer Publishing and Ivyspring International Publisher.

Orthotopic 9L gliosarcoma rat models were used to monitored AGuIX® NP biodistribution by MRI after iv injection and to accurately quantify the amount of UNPs at the tumor site. In this model, AGuIX® NPs accumulate at the tumor within the first minutes after iv injection, reaching the maximal amount at 15-60 min. Moreover, UNP elimination from the contralateral hemisphere is much faster compared to the tumor site in which the UNPs can still be detected by MRI 24 h p.i.. As already observed with the commercial MRI contrast agent Dotarem®, AGuIX® NP is able to cross the blood-brain barrier in brain cancer models which highlights the disruption of this barrier at the vicinity of the tumor site. The delay to wait for treating animals upon RT is determined at the optimal Gd content ratio between the tumor and the surrounding tissues. In a first study, the animals were treated 10 days and 17 days after tumor implantation and the irradiation of the whole brain (10 Gy) was performed 7 h after iv AGuIX® NP injections (100 µmol of Gd^3+^, 500 mg/kg) since no Gd could be detected by MRI in the contralateral area at this time (tumor to contralateral ratio of 14.52) (Figure 2A) 36. The Gd concentration in the tumor was computed from the longitudinal relaxation time T_1_ value measured by MRI (T_1_ is inversely proportional to the Gd concentration). The Gd concentration in the tumor was reaching 227.9 ± 60 µM and 29.8 ± 8.3 µM at 1 h and 7 h p.i. respectively (Figure 2A). The RT treatment combined with AGuIX® NPs induced a tumor volume reduction of 26% compared with the RT group at day 17.

Another study in the same model provided an accurate determination of Gd content and location by *ex vivo* X-ray fluorescence (XRF) map 39. Gd content in the whole brain was going from 550 to 15 ppb between 1 and 24 h after iv injection (56 µmol of Gd^3+^). A comparison of irradiation (X-rays from synchrotron) at 1 and 24 h after iv injection was performed. Unexpectedly, median survival time (MeST) were 62 and 95.5 days and increase in life span (ILS) were 210% and 378% at 1 h and 24 h respectively (*vs* 20 days and 130% with RT only) which revealed that lower Gd concentration was inducing higher therapeutic effect (Figure 2B). This apparent paradoxical result can be explained in two different ways. First, the Gd content in the tumor 24 h after injection is sufficient to induce a therapeutic effect upon RT while the NPs are entirely wash-out from the outer part of the brain 24 h after injection. This is not the case 1 h after injection (Figure 2A) which could induce toxicity in the non-affected area. Secondly, the distribution of Gd observed by XRF showed to be very different at 1 and 24 h after iv injection with a broad dissemination at 1 h and a more localized one at 24 h. This suggests that UNP localization in the tumor could play an important role. These results outline the crucial role of EPR effect inducing an accumulation and retention of UNPs at the tumor site leading to both complete washouts of the UNPs from the contralateral area and potentially a different repartition of UNPs into tumor for higher therapeutic outcomes. Importantly, the MeST of the rat model treated by AGuIX® NP injection (100 µmol) without RT and the non-treated animals revealed the same MeST profile, confirming the absence of toxicity of AGuIX® NPs without RT which is a key feature to go forwards to human.

Dotarem® is a Gd-chelate based MRI contrast agent which has been used in patients for more than 20 years. Thanks to the presence of Gd^3+^ ion in the structure of Dotarem®, a radiosensitizing effect could be expected during RT treatment. A therapeutic efficacy comparison of the AGuIX® NP (~10 kDa) with the small molecule Dotarem® (557 Da) on the same model upon RT (X-rays from synchrotron) 20 min after iv injection (56 µmol of Gd^3+^ for both) was performed 40. The short delay of 20 min was selected to maintain a relatively high concentration of Dotarem® in the tumor during irradiation. No or low impact of Dotarem® was observed on the survival curve (MeST are 32 and 43 days, corresponding to ILS of 68% and 126% relative to the non-treated group, for injection concentrations of 1 M and 40 mM respectively) compared to the radiation-only treatment (MeST 44 days, ILS 131%), whereas high therapeutic efficacy of the AGuIX® NP (injected at 40 mM) was observed (MeST 102.5 days and ILS of 439%) (Figure 2C). These different therapeutic outcomes can be explained by (i) different biodistribution profiles between Dotarem® and AGuIX® NP and (ii) nanoscale energy depositions provided by the co-activation of neighbouring Gd atoms gathered into a nanoplatform leading to dose-effect increase to several orders of magnitude 45.

Studies on the AGuIX® NP biodistribution were also performed with a brain melanoma metastases model (B16F10) 41. Intravital two-photon microscopy on a sub-cutaneously implanted model showed an increase of labelled-AGuIX® NP accumulation from 1 to 3.5 h after iv injection, and a decrease at 24 h but with relatively high persistence (21% compared with that at 3.5 h) (Figure 2D). The corresponding orthotopic cancer model (implantation in brain) treated by RT (7 Gy, 95% of the brain covered by irradiation) 3.5 h after AGuIX® NP iv injection (1.2 µmol of Gd^3+^) showed an ILS to 25% (*vs* 8.3% with RT only) (Figure 2E).

**Figure 3:**
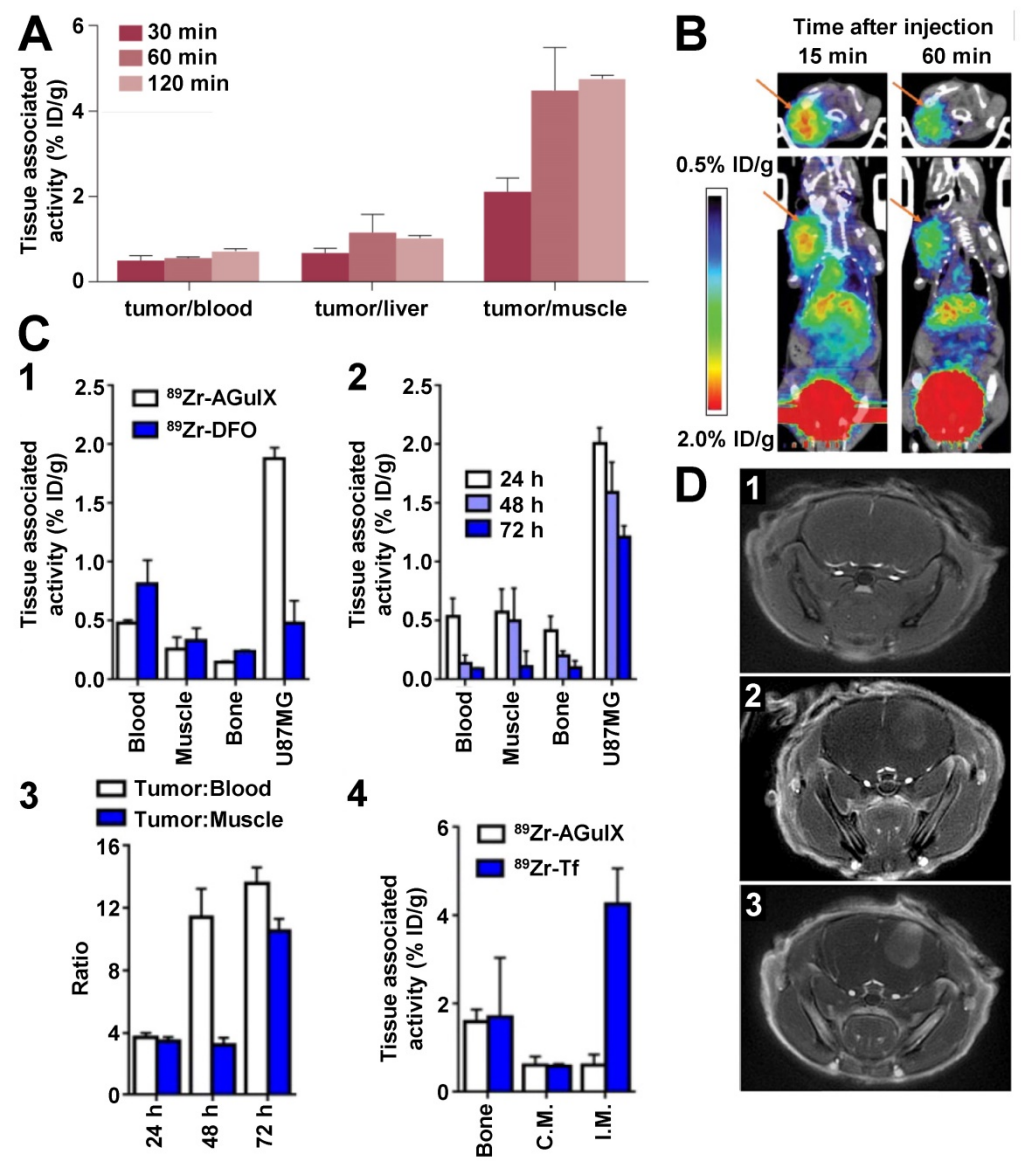
**Studies of AGuIX® NPs on glioblastoma rodent cancer models. A.** Tumor-to-tissue ratios of ^68^Ga-NODAGA-AGuIX at 30, 60 and 120 min p.i. in U87MG tumor-bearing mice. **B.** PET images of a U87MG tumor-bearing mouse injected with ^68^Ga-NODAGA-AGuIX, at 15 and 60 min p.i.. **C.** ^89^Zr-DFO-AGuIX NPs accumulate in the tumor microenvironment, but not in inflammatory abscesses. **C1.** Biodistribution data at 24 h p.i. of ^89^Zr-DFO-AGuIX NP or ^89^Zr-DFO show significantly higher uptake of the UNPs in the microenvironment of subcutaneous U87MG tumors compared to ^89^Zr-DFO. No substantial differences were observed in muscle or bone, two normal reference tissues, from the cohorts receiving either radionuclide. **C2.** Biodistribution data showing that ^89^Zr-DFO-AGuIX NPs persist in the tumor microenvironment for several days p.i.. At 72 h p.i., the tumor associated activity was ∼1.0% ID/g, which is above background. **C3.** A graphical representation of the mean tumor to muscle and tumor to blood ratios over time for mice treated with ^89^Zr-DFO-AGuIX NPs. **C4.** Biodistribution data showing no uptake of ^89^Zr-DFO-AGuIX NPs in the inflamed muscles within the hindlimbs of a mouse cohort. By comparison, ^89^Zr-transferrin (Tf) showed robust uptake in the inflamed muscle, presumably owing to the abundant expression of the transferrin receptor on peripheral mononuclear blood cells. C.M. = contralateral unmanipulated muscle; I.M. = inflamed muscle. **D.** MR axial image of the brain before (1) and after orotracheal (ot) (2) or iv (3) administration of the AGuIX® NPs; (2) was acquired approximately 30 min after ot administration of 50 μL UNP (200 mmol/L [Gd^3+^]) whereas (3) was acquired approximately 30 min after iv administration of 200 μL UNP (50 mmol/L [Gd^3+^]). Adapted with permission from ref 42-44, copyrights 2019 John Wiley and Sons, American Chemical Society and Future Medicine Ltd.

Glioblastoma mouse models (sub-cutaneously implanted U87MG cells) have been used to assess biodistribution of the modified-AGuIX® NP for dual-modality PET/MRI detection. PET has the advantage to be much more sensitive than MRI and radiolabelled NPs can accurately be quantified by *ex vivo* autoradiography. ^68^Ga has a physical half-life of 68 min which enables imaging during one to two hours after production depending on the injected radiopharmaceutical dose. ^68^Ga isotope was introduced on AGuIX® NP by grafting of NODAGA (1,4,7-triazacyclononane,1-glutaric acid-4,7-acetic acid-1,2-diaminoethane) chelates at the surface 44. *Ex vivo* autoradiography of tumor extracted from the mouse models showed that intravenously-injected ^68^Ga-modified AGuIX® NPs (6.8 µmol of Gd^3+^/27 µCi) passively accumulate into the tumor at 30 min p.i. (1.03 ± 0.11% ID/g) and remains stable at 1 h p.i. (1.10 ± 0.16% ID/g). The tumor uptake dropped from 1 to 2 h p.i., however, the tumor/blood and tumor/muscle ratios increased over time, with the last one reaching almost 5 at 2 h p.i. (Figure 3A). *In vivo* PET imaging confirmed the detection of the intravenously-injected ^68^Ga-modified AGuIX® NPs (10.2 µmol of Gd^3+^/180 µCi) enabling clear delineation of the tumor 1 h p.i. (Figure 3B). This study also outlined the importance of the AGuIX® NP nanostructure for retention into the tumor since the AGuIX® NPs injected in lower amount (0.5 µmol of Gd^3+^) was rapidly washed out from the tumor and metabolite studies revealed 90% degradation at 5 min p.i. due to the UNP dilution. While proton MRI and ^68^Ga radiolabelling can hardly be used to detect NPs in the rodent 24 h p.i., the introduction of ^89^Zr through desferrioxamine (DFO) chelate grafting at the surface of AGuIX® NPs was used to monitor the UNP biodistribution over 72 h p.i. thanks to the relatively high half-life of 3.3 days of the radioisotope 43. This study revealed an accumulation of ^89^Zr-modified AGuIX® NPs (20 µmol of Gd^3+^/50 µCi) at the tumor site 20 min p.i. and a durable retention from 24 to 72 h (1-2% ID/g) exhibiting tumor/blood and tumor/muscle ratios greater than 10 at 72 h after iv injection, whereas the control small chelate compound ^89^Zr-DFO was less accumulated at the tumor site (0.5% ID/g 24 h p.i.) (Figure 3C). Moreover, ^89^Zr-modified AGuIX® NP accumulation was not observed in inflammatory abscesses (obtained by intramuscular injection of 50 µL turpentine) confirming that UNP accumulation requires the tumor-associated EPR effect to take place. In contrast, the control protein ^89^Zr-modified-transferrin could successfully target the inflammation area by targeting the receptors associated to the acute phase response.

Interestingly, orotracheal (ot) administration of the AGuIX® NPs (10 µmol of Gd^3+^) in an orthotopic brain mouse model (U87) showed a slower accumulation of the UNPs at the tumor site (20-30 min *vs* 5-10 min after ot and iv injection respectively) and an elimination time from the tumor about 70% longer than with iv injection of the same amount of AGuIX® NPs (Figure 3D) 42. The amount of orotracheally-administrated UNPs into the tumor site is lower than with iv injection at any time p.i., however the delayed accumulation and elimination suggest a longer residential time at the tumor site for the orotracheally-administrated UNPs. This study demonstrated that the ot route is effective to passively accumulate UNPs into tumors through a favoured traffic across the alveolar surface and EPR effect once in the blood. The different pharmacokinetic profile obtained by ot administration could provide efficient passive targeting strategy.

## Preclinical studies on other types of tumors

Accumulation and retention of the AGuIX® NPs have also been observed in other types of rodent cancer models such as pancreatic 38, hepatic colorectal 46-47 and lung 48-49 cancer models.

**Figure 4:**
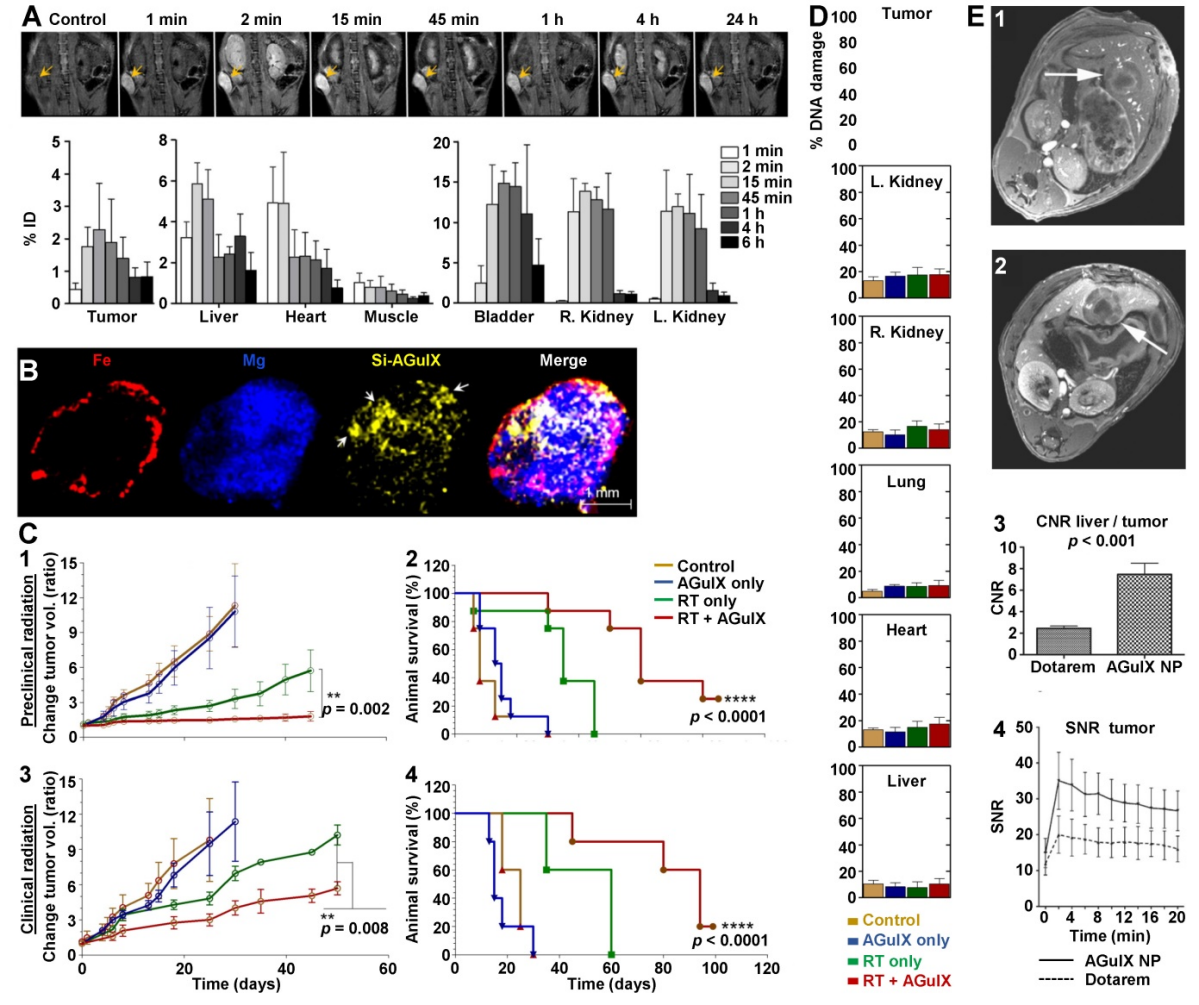
**Studies of AGuIX® NPs on pancreatic and hepatic colorectal rodent cancer models. A.** Whole body MRI and blood plasma kinetics in mice. T_1_-weighted MRI (7 T) of AGuIX® NPs (0.25 mg/g) injected in mice bearing capan-1-pancreatic tumors (n = 3/group) showed early tumor discrimination (at 1 min p.i.) followed by increasing accumulation. Maximum tumor accumulation of AGuIX®NPs occurs at 15 min p.i.. Some accumulation in the liver (~6% ID) was also observed. All data are represented as the means ± standard deviation. **B.** Intratumoral AGuIX® NP localization in capan-1 tumor at 15 min p.i. imaged using LIBS. **C.** Tumor volume measurements (1, 3) and therapeutic effect assessments (2, 4) on capan-1-tumor-bearing mice treated with and without AGuIX® NP and preclinical (220 kV, n = 8/group, 1, 2) or clinical (6 MV, n = 5/group, 3, 4) radiation (10 Gy). The tumor volume is significantly decreased and the Kaplan-Meier survival curves demonstrate significant survival benefit when AGuIX® NP is included with both preclinical and clinical radiation. Statistical significance was calculated using the log-rank (Mantel-Cox) test. **D.** Preclinical radiation-induced DNA damage studies. γH2AX+ nuclei were counted across multiple image planes (n = 50) and further quantified. The data are represented as the means ± standard deviation. ****: *p* < 0.0001. **E.** T_1_‐weighted axial image in a rat with a hepatic colorectal cancer metastasis (diameter approximately 8 mm, arrows) after administration of Dotarem® (1) and AGuIX® NP (2) (0.1 mmol/kg [Gd^3+^] in both case). Post‐contrast images with AGuIX® NP better depict the mass relative to Dotarem® as is evident from the greater contrast‐to‐noise ratio (CNR, 3) between normal liver tissue and the metastasis. AGuIX® NP demonstrates kinetics comparable to a low‐molecular contrast agent (4). SNE: signal-to-noise ratio. Adapted with permission from ref 38, 46, copyrights 2019 Elsevier and John Wiley and Sons.

Pancreas models are known to exhibit a low vascularization associated with a dense ECM which may limit accumulation owing to the EPR effect 50. The AGuIX® NPs (0.25 mg/g) were injected in subcutaneous-capan-1-tumor bearing mice and the quantitative monitoring of the UNPs in different organs after iv injection was achieved by MRI 38. A progressive accumulation of AGuIX® NPs was observed from 1 min p.i., reaching a maximal MRI signal in the tumor 15 min p.i. (2.27 ± 0.44% ID) (Figure 4A). The early clearance of the UNPs was confirmed by a concomitant prominent signal at 15 min p.i. in the kidneys and bladder (right kidney: 13.85 ± 0.98% ID; left kidney: 11.92 ± 1.58% ID; bladder: 14.85 ± 1.49% ID). *Ex vivo* imaging by laser-induced breakdown spectroscopy (LIBS) confirmed the presence of the UNPs in the tumor (Figure 4B, Si signal) and revealed a heterogeneous distribution attributed to the higher vascularity observed at the periphery of the tumor compared to the core (Figure 4B, Fe signal). Treatment of pancreatic cancer mice upon both preclinical (small animal radiation research platform (SARRP), 220 kVp) and clinical (6 MV) irradiation sources (10 Gy) was performed 15 min after AGuIX® NP iv injection (0.25 mg/g). The tumor volume and animal survival were both significantly different in the treated *vs* non-treated groups upon preclinical and clinical irradiation sources (Figure 4C). Study of radiation-induced DNA damage (γH2AX staining) was performed by *ex vivo* imaging. The *in vivo* treated tumors showed a much higher DNA damage in the UNP-irradiation-treated cohort with more than 80% DNA damage compared to about 60% for the radiation-only group and to lower than 10% for the other control groups (Figure 4D). This study clearly highlights that EPR-mediated accumulation into the tumor is well adapted for UNPs whose therapeutic action is specifically triggered by RT. This dual-targeting concept is very effective to overcome the current limitation of low drug accumulation owing to the EPR effect.

EPR-mediated accumulation of the AGuIX® NPs was also demonstrated in hepatic colorectal rat cancer models 46-47. AGuIX® NP and Dotarem® iv injections were compared (0.1 mmol/kg of Gd^3+^ for both). The tumor detection by MRI was much easier using the UNPs due to a greater contrast-to-noise ratio (CNR) between normal liver tissue and liver metastases (Figure 4E). Similar kinetics were observed with a strong peak enhancement in the tumor just after iv injection followed by continuous washout within the first 20 min p.i..

**Figure 5:**
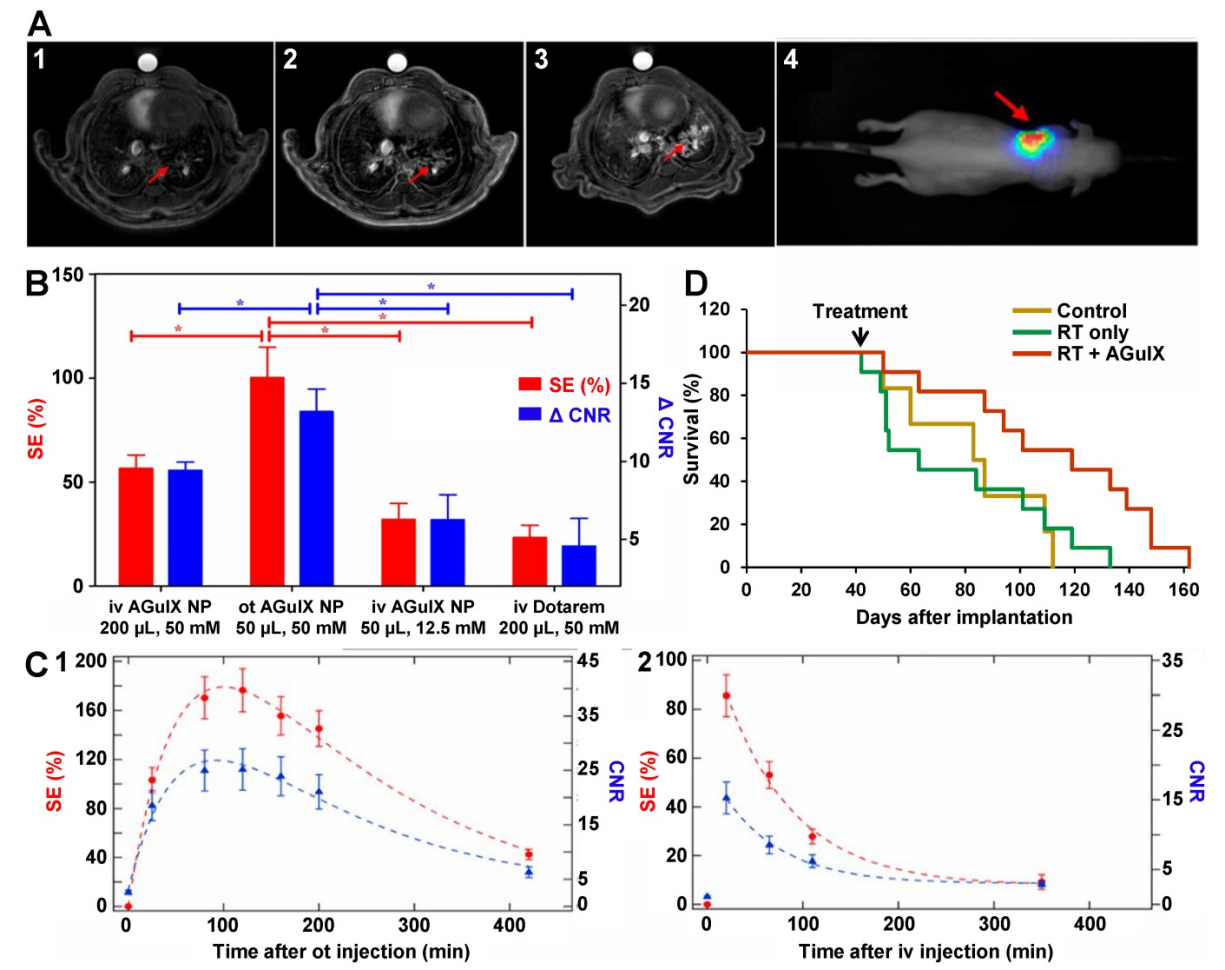
**Studies of AGuIX® NPs on lung cancer rodent model. A.** Ultrashort echo time MRI axial slices of the H358‐Luc lung tumor-bearing mice before (1) and after iv administration of 200 μL of 50 mM [Gd^3+^] AGuIX® NP (2) or ot administration of 50 μL of 50 mM [Gd^3+^] AGuIX® NP (3). The presence and colocalization of the tumor was assessed with BLI (4). In (4) the scale colors are proportional to the number of detected photons per second. The arrow shows the tumor in the left lung. **B.** Bar plot comparing the SE (red, left scale) and the ΔCNR (blue, right scale) for ot and iv administration of AGuIX® NP and Dotarem®. *: *p* < 0.05. Data are expressed as the means ± standard error of the mean. The ΔCNR was computed as the difference between the CNR before and after the administration of AGuIX® NP or Dotarem®. **C.** Curves of SE using ultrashort echo time MRI (red, left scale) and CNR (blue, right scale) after ot (50 μL of 50 mmol/L [Gd^3+^]) (1) or iv (200 μL of 50 mmol/L [Gd^3+^]) (2) administration of AGuIX® NPs. These graphs show the different pharmacokinetics observed after the two administration routes. Dashed lines exhibit the curves trend. Data are expressed as the means ± standard error of the mean. **D.** Survival curve comparison of tumor‐bearing mice without treatment (n = 6), only treated by irradiation (n = 11), and treated by irradiation (n = 11) 24 h after ot administration of AGuIX® NPs, 161 days after tumor implantation. The irradiation was performed at 10 Gy, 37 days after tumor implantation. Adapted with permission from ref 48-49, copyrights 2019 John Wiley and Sons and National Academy of Sciences.

Passive targeting of AGuIX® NPs on lung cancer models was confirmed in several studies 48-49. Accumulation of both intravenously and orotracheally-administrated AGuIX® NPs in an orthotopic luciferase-modified non-small-cell lung cancer (NSCLC) mouse model (H358-Luc cells) was validated by *in vivo* bioluminescence imaging (BLI) and MRI (Figure 5A) 48. Interestingly, signal enhancement (SE) and CNR in the tumor were about twofold higher after ot administration (2.5 µmol of Gd^3+^) compared to iv injection (10 µmol of Gd^3+^) of the UNPs using four times less Gd amount (Figure 5B). *Ex vivo* histological analyses (fluorescence reflectance imaging) showed that the Cy5.5 modified-AGuIX® NPs were visible in the tumor up to 72 h after both types of administration. Nevertheless, the pharmacokinetic of the UNPs in the tumor was different with the type of administration route (Figure 5C). Indeed, the maximum SE (50-110 min *vs* 10-20 min) and the elimination (50 % drop of the SE at 235 ± 56 min *vs* 62 ± 12 min) were both delayed after ot administration. This unexpected behaviour observed after ot administration was explained by both EPR effect and a direct tumor targeting of the UNPs once in the alveoli due to the very thin alveolar barrier. The lung tumor-bearing mice exposed to conventional irradiation (10 Gy) 24 h after ot administration of AGuIX® NPs (1 µmol of Gd^3+^) showed MeST much extended (112 days) compared to the non-treated (83 days) and irradiated-only (77 days) groups, which corresponds to 45% increase in ILS (Figure 5D) 49.

## Clinical trials on brain metastases

AGuIX® NP accumulation into tumor tissues by EPR effect was validated in several rodent models, and the UNP retention into the tumor up to several hours or days revealed to be very valuable for the therapeutic outcomes. Nevertheless, this passive targeting strategy still has to be confirmed in human since the complex heterogeneity of the tumor types and environments impacts the EPR effect efficacy and its translation into clinical application is currently controversial 5, 51.

**Figure 6:**
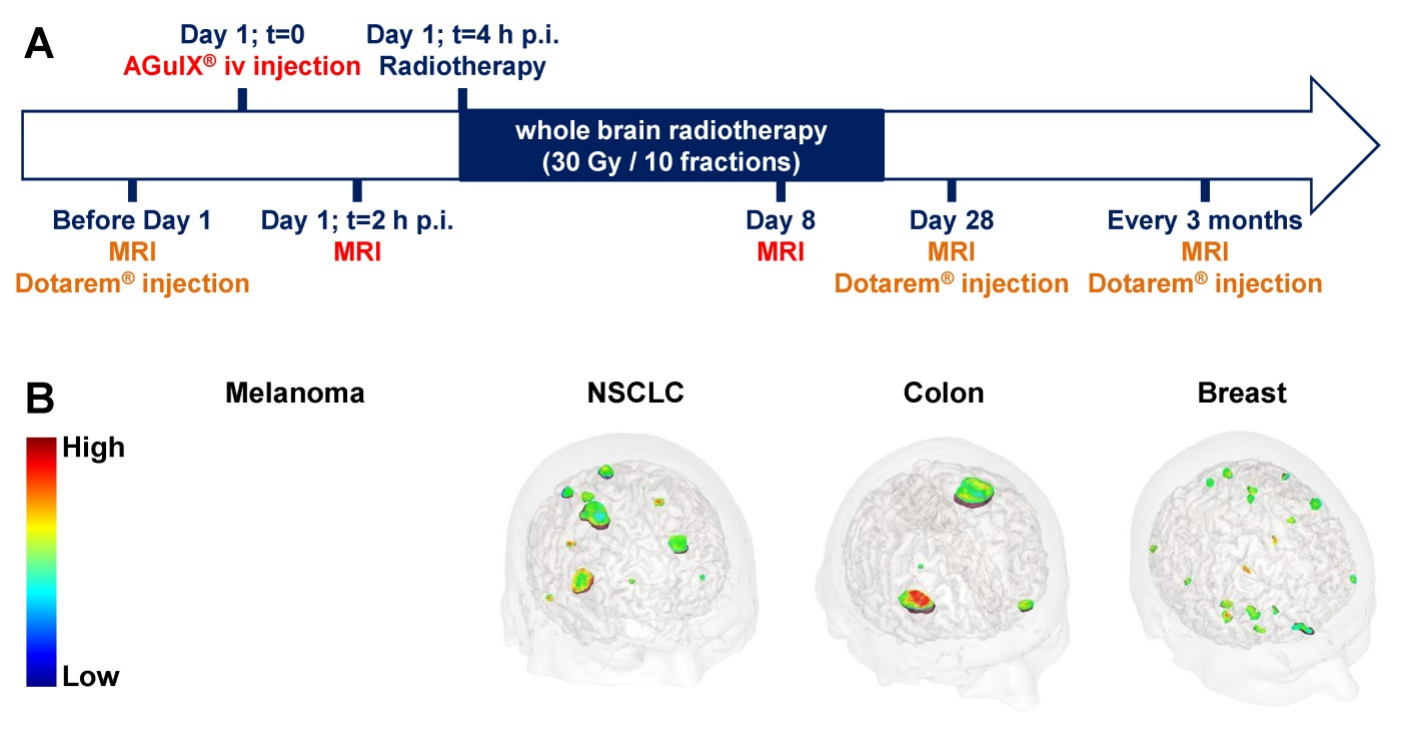
**Phase Ib clinical trial assessing AGuIX® NP radiosensitization. A.** Protocol for the clinical trial of phase Ib (NCT03308604, NANORAD) to assess radiosensitization of multiple brain metastases using AGuIX® NP. This first-in-man clinical trial aims at studying the tolerance of AGuIX® NP iv administration in combination with whole brain radiation therapy and determining the recommended dose of AGuIX® NP for phase II clinical trial. **B.** 3D MRI from patients included in the NANORAD clinical trial obtained 2 h after iv injection of AGuIX® NPs. The brain metastases stemming from the four different types of primary cancers (melanoma, non-small cell lung cancer (NSCLC), colon cancer and breast cancer) were targeted by the AGuIX® NPs while no enhancement of the MRI signal was observed in healthy tissues. The patients were treated with 15 to 100 mg/kg of AGuIX® NPs (dose escalation). For the brought forward MRI data, the patients with melanoma, NSCLC, colon cancer and breast cancer received 15, 50, 50 and 75 mg/kg doses of AGuIX® NP respectively. Adapted with permission from ref 30, copyright 2019 BIR Publications.

Thanks to its safety related to fast renal clearance coupled to non-toxicity in the absence of radiation, the AGuIX® NPs are currently assessed in two clinical trials of phase Ib for radiosensitization in patients affected by multiple brain metastases 34-35 and by locally advanced cervical cancer 52. These UNPs have recently been approved for a clinical trial of phase II for the multiple brain metastases indication (NANORAD2) 53. We present herein preliminary data from the first-in-human clinical trial of AGuIX® NP for radiosensitization in patients suffering from multiple brain metastases and relate them with accumulation and retention of the AGuIX® NPs into the tumors. The patients (n = 15), with multiple brain metastases from melanoma, lung cancer (NSCLC), colon and breast primary tumors, were recruited for whole brain radiation therapy when they were ineligible for local treatment by surgery or stereotactic radiation. Theranostic NPs combining both diagnostic and therapeutic properties are very valuable for clinical investigation, and especially for validating accumulation and retention into the tumor owing to the EPR effect. This clinical trial was designed in order to take advantage of the MRI detection of AGuIX® NPs to withdraw biodistribution information in addition to the maximal tolerated dose (Figure 6A). Indeed, after a pretherapeutic standard brain MRI (with Dotarem® injection) to localize the tumors, the patients were iv injected with the AGuIX® NPs (15, 30, 50, 75 or 100 mg/kg) at D1 2 h before performing brain MRI exam (T_1_ weighted-images) to confirm the UNP accumulation at the tumor sites. Then the patients underwent a whole brain radiation therapy (30 Gy in 10 sessions) and brain MRI exams were performed at D8, and then at D28, 3 months and every 3 months up to one year of follow-up (with Dotarem® injection for the follow-up sessions). As for preclinical studies, the irradiation protocol was modelled on the standard procedure used in the current treatment. Even if MRI exams at D8 was not a standard approach in clinical trial, the theranostic properties of the AGuIX® NPs incited us for this monitoring to gain insight into toxicity and retention of the UNPs in the brain metastases.

A significant MRI SE was clearly observed at D1, 2 h after AGuIX® NP iv injection for all types of brain metastases and all assessed doses (15 to 100 mg/kg) (Figure 6B). Interestingly, SE in metastases increased with increasing the injected AGuIX® NP dose 54. The mean UNP concentrations at the tumor sites determined by MRI was comparable to the concentrations observed in preclinical studies.

**Figure 7:**
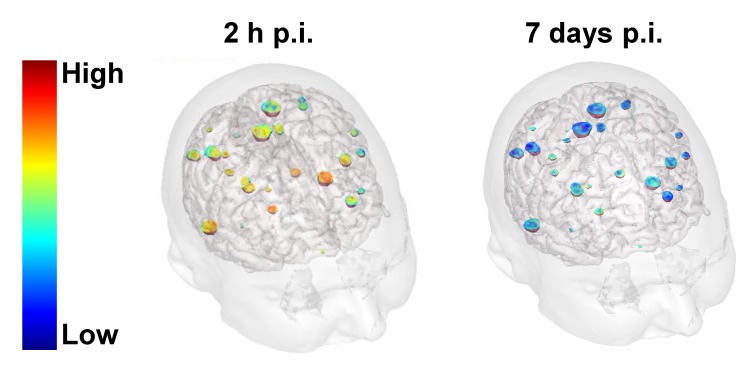
** Validation of AGuIX® NP retention in tumors in human 7 days after iv injection.** 3D MRI from one of the patients (NSCLC primary cancer) treated with the highest dose of AGuIX® NPs (100 mg/kg) obtained 2 h and 7 days after iv injection. The 3D MRI confirmed that SE was still detected in the brain metastases 7 days after iv injection of AGuIX® NPs.

Moreover, brain MRI exams carried out at D8 revealed retention of the AGuIX® NPs at the tumor sites in the three patients injected with the highest dose (100 mg/kg) (Figure 7). This observation unambiguously confirms that accumulation and retention of the AGuIX® NPs at the tumors sites were successfully translated from preclinical models to patients.

## Discussion on EPR effect of UNP

Investigations for cancer treatment over the last 10 years using the theranostic AGuIX® NP (4 ± 2 nm HD, ~10 kDa) led us to reconsider the described limitations of EPR-mediated tumor targeting 30. It is worth to note that the diagnostic property of the AGuIX® NP has been very valuable to confirm its *in vivo* biodistribution by MRI in rodent and non-human primate models and more importantly in human during phase Ib clinical trial.

The first dogma fixes a lower size threshold of NPs at about 40 kDa to favour a high NP concentration in plasma required for a long blood circulation (> 6 h), which showed to improve accumulation owing to the EPR effect 51. However, we observed in several preclinical studies accumulation and retention of much lower-size UNPs at the tumor site over 3 days in rodent cancer models. EPR effect was also observed in a pancreatic cancer rodent model known for limited drug access. Moreover, accumulation and retention of the AGuIX® NPs was validated by MRI in human during a phase Ib in patients with multiple brain metastases. Therefore, the size threshold to differentiate small molecules and NPs for their EPR efficacy could be lower to about 10 kDa (2-6 nm), as suggested by the retention differences in tumors observed from Dotarem® (0.5 kDa) and AGuIX® NPs over one to three days and seven days after iv injection in preclinical and clinical studies respectively 40, 43. Indeed, accumulation and retention of NPs in tumor are complex processes depending on several properties such as shape, hardness, charge density and hydrophobicity of NPs, which can modulate the size threshold. Most of the intravenously-injected drugs would tend to reach the tumor site as long as the tumor is well vascularized. Accumulation at the tumor site just after iv injection (few minutes to hours) would be expected as it is the case for several widely used small molecules as PET or MRI-tracers (such as the fluorine-18 fluoro-deoxy-glucose ([18F]FDG) or Dotarem®). However, small molecules usually exhibit fast washout (from minutes to few hours) from the tumor, whereas UNPs can be retained for much longer. The ultrasmall size seems to be well adapted for fast accumulation and relatively long retention time at the tumor site as well as fast elimination from blood circulation thanks to renal filtration.

The low dose of drug accumulated at the tumor site, approaching 0.5-3% ID depending on the tumor environment and drugs used, is often pointed out as a limitation of the EPR effect. Nevertheless, the most important aspect to consider is the damage induced by the drugs targeted into the tumor over the drugs in other organs and especially those most susceptible to accumulate the drugs such as the kidneys, the spleen, the liver and the lungs, depending on the drug-elimination route and drug size. In the optimal case, the drug should be exclusively toxic when located into the tumor which can be achieved if the therapeutic actions are triggered by endogenous-tumor specific or external stimuli. In such a way, even very low dose of the accumulated drugs could be sufficient to induce a therapeutic effect without toxicity in other organs. In the case of the AGuIX® NP, the therapeutic effect is triggered by RT which can be applied to localized areas. The irradiation is performed after the complete elimination of the UNPs from the blood and healthy part of the irradiated organ(s) whereas the tumor still contains some UNPs thanks to the accumulation and retention obtained owing to the EPR effect. Even very low AGuIX® NP concentration in the ppb range proved to induce significant therapeutic outcomes 39 which make EPR-mediated targeting strategies highly valuable. The therapeutic effect at such low UNP dose can be explained by the nanoscale energy depositions which increase the RT dose effect in a very close vicinity (< 10 nm) of the UNPs to several orders of magnitude thanks to the nanostructure of the drug 45. Moreover, external stimulus-activable UNPs open the route for fractionated treatment especially if retention at the tumor site is long enough. For cancer treatment, radiation fractionation is a standard procedure thanks to the lower capacity of tumor cells to recover from low radiation dose compared to healthy cells.

We observed over several preclinical studies and clinical trial using AGuIX® NP that cancer targeting by the passive EPR effect can be very efficient to achieve therapeutic outcomes. Nevertheless, some key features were required to take advantages of EPR-mediated cancer targeting. First, (i) only the UNPs localized into the irradiated area (containing the tumors) induce a therapeutic action, (ii) a low concentration of UNPs into the tumor (ppb range) provides therapeutic effect and (iii) the small size of UNPs was suitable for fast elimination from blood circulation thanks to renal clearance reducing any risk of toxicity in the non-irradiated organs. MRI-detection of the UNP is another useful but not required property which helps to validate EPR-effect efficacy and to determine the optimal time to trigger the therapeutic action upon RT.

## Conclusion

Achieving tumor targeting to improve therapeutic effect and reduce the adverse ones is a major challenge in cancer treatment. In spite of some scepticism raised from passive targeting owing to the EPR effect, we experienced that such targeting strategy can be very valuable for UNPs whose therapeutic action is efficient at low dose and specifically activated at the tumor site by an external stimulus. The works carried out with the AGuIX® NP reveal how EPR effect induced accumulation and more importantly retention of the UNPs at the tumor sites which is crucial for higher therapeutic outcomes. This therapeutic strategy based on passive accumulation and retention at the tumor sites was validated in several preclinical models and in a clinical trial of phase Ib for radiosensitization in patients affected by multiple brain metastases.

## Abbreviations

AGuIX®: activation and guiding of irradiation by X-ray
ANSM: *Agence nationale de sécurité du médicament et des produits de santé*
αMSH: alpha melanocyte stimulating hormone
BLI: bioluminescence imaging
C dot: Cornell dot
CFCT: corresponding normalized cell fluorescence
C.M.: contralateral unmanipulated muscle
CNR: contrast‐to‐noise ratio
DFO: desferrioxamine
DOTAGA: 1,4,7,10-tetraazacyclododecane,1-glutaric acid-4,7,10-triacetic acid
ECM: extracellular matrix
EPR: enhanced permeability and retention
FDG: fluoro-deoxy-glucose
HD: hydrodynamic diameter
HER2: human epidermal growth factor receptor 2
ID: injected dose
IFP: interstitial fluid pressure
ILS: increase in life span
I.M.: inflamed muscle
iv: intravenous
LIBS: laser-induced breakdown spectroscopy
MeST: median survival time
MRI: magnetic resonance imaging
MRT: microbeam radiotherapy
NODAGA: 1,4,7-triazacyclononane,1-glutaric acid-4,7-acetic acid-1,2-diaminoethane
NP: nanoparticle
NSCLC: non-small-cell lung cancer
ot: orotracheal
PET: positron emission tomography
p.i.: post-injection
RT: radiotherapy
SARRP: small animal radiation research platform
SNE: signal-to-noise ratio
Tf: transferrin
UNP: ultrasmall nanoparticle
XRF: X-ray fluorescence
